# Quality of life of obstetrics fistula patients before and after surgical repair in the Jimma University Medical Center, Southwest Ethiopia

**DOI:** 10.1186/s12905-021-01360-y

**Published:** 2021-05-21

**Authors:** Tilahun Fufa Debela, Zerihun Asefa Hordofa, Aster Berhe Aregawi, Demisew Amenu Sori

**Affiliations:** 1 Department of Health Policy and Management, Faculty of Public Health, Jimma University, Jimma, Ethiopia; 2 Maternal Health Project Coordinator at Jimma University, Jimma, Ethiopia; 3 Program Analyst, Midwifery United Nations Population Fund, Addis Ababa, Ethiopia; 4 Department of Obstetrics and Gynecology, Jimma University, Jimma, Ethiopia

**Keywords:** Fistula, Obstetric, Quality of life, Preoperative, Postoperative, Jimma

## Abstract

**Background:**

The consequences of obstetric fistula on affected women are more than the medical condition. It has extensive physical, psychological, social, and economic consequences on them. Obstetric fistula affects the entire health and entire life of women. Women suffering from obstetric fistula are often abandoned by her partner, relatives, and the community. This study aimed to determine the quality of life of obstetrics fistula patients before and after surgical repair.

**Methods:**

Institutional-based prospective, before and after study design was conducted in the Jimma University Medical Center from November 1, 2019–October 30, 2020. A face-to-face interview was conducted with fistula patients who visited Jimma University Medical center, fistula clinic during the study period. All fistula patients were included in the study. Accordingly, 78 women who underwent surgical repair were interviewed. The means and the standard deviation were computed using conventional statistics formulas. The unpaired t-test was used to compare two independent means, and one-way analysis of variance (ANOVA) was used to compare the quality of life before repair and after a successful repair. Linear regression analysis was done for identifying determinants of quality of life. A *P* value of 0.05 will be considered statistical significance.

**Result:**

The overall quality of life of women was 58.17 ± 7.2 before the surgical repair and 71.20 ± 10.79 after surgical repair. The result indicates there is a significant difference in the mean value of pre and post-operative (*P* < 0.001). The overall satisfaction of women with their health status before the surgical repair was 22.5 ± 1.30and it has increased to 53.0 ± .90after surgical repair. The physical health dimension score was 16.51 ± 5.27 before the surgical repair while it has increased to 21.77 ± 5.38 after the surgical repair. The score of the social domain before the surgical repair was 5.19 ± 1.34 and it has increased to 7.13 ± 3.67 after the surgical repair. The score of the environmental health domain was 17.41 ± 2.89 before the surgery while it also increased to 21.65 ± 4.04 after the surgical repair. The results have shown there was a significant difference in the mean values of pre and post-operatives in both social and environmental scores (*P* < 0.001). The score of the psychological health domain before the surgery was 19.06 ± 1.46 and it was increased to 19.84 ± 3.21 after the surgical repair. The result showed there is a significant difference in mean value pre and post-operative (*P* = 0.048), though it is a slight improvement compared to other domains.

**Conclusion:**

The overall quality of life of the patient with fistula was improved after successful surgical repair. Although all domains of quality of life had shown significant improvement after successful surgical repair, the psychological domain showed slight improvement.

## Background

Globally, more than 2 million women are living with obstetric fistula [1]. In Sub Sahara Africa, obstetric fistula is estimated that 1.60 cases per 1000 women of reproductive age, and in South Asia the prevalence is estimated at 1.20 fistula cases per 1000 reproductive age women [2]. Obstetric fistula is a serious and devastating complication of childbirth affecting millions of women in the world, especially the developing world.

An obstetric fistula refers to an abnormal opening between a woman's genital tract and her urinary tract or rectum produced by obstetric causes, usually prolonged and obstructed labor [3]. Fistula affects usually women from rural areas, who are poor, uneducated women and often with early first pregnancy [4]. Unfortunately, women who endure such obstructed labors and resulting incontinence are often young, undernourished, and married early [5]. The obstetric fistula is one of the most common and certainly most dreaded injuries [5]. The impact of fistula in a country where maternal mortality rates are high is countless [6].

Obstetric fistula has an extensive physical, psychological, social, and economic cost for women affected [7]. The women are left with persistent incontinence and the baby inevitably dies due to prolonged obstructed labor [8]. As a result of fistula, women can't control the flow of urine or feces or both. This may lead to abandonment by her partner, relatives, and /or detestation by the community [9]. If treatment is not given to such victimized women, the life of the women and her family fall in danger [4]. Several women are divorced or abandoned by their husbands, turned back by friends, and even by health professionals [10].

Obstetric fistula is not only devastating the physical health of women but emotionally and socially. Fistula is a massive psychological disturbance [11]. It leads to severe sociocultural stigmatization. In developing countries, obstetric fistula is always linked to prolonged labor and poorly performed abortion [4]. The Ethiopian health system suffers from the burden of other public health issues like malaria, HIV/AIDS, and tuberculosis breakdown [12]. This may put many women at risk. Our Hospitals suffer from shortages of supply and manpower especially highly skilled professionals who can do the surgical repair for women with obstetric fistula [13]. There is also low institutional delivery in the country, in which women give birth in their home [14]. The other challenge related to the fistula is the lack of information. Most women do not know as there is a treatment for fistula [15]. They hide their circumstances and be ill within silence [11]. Besides, the treatment of fistula is among the type of expensive treatment. The treatment center needs a laboratory, radiology, and blood bank before conducting the surgery to know patient history [15]. After surgery, it is needed post-operative wards, anesthetic services, psychological and social integration [3]. The cost of salary, single used medical equipment, and other infrastructure, together endow with huge economic loads to treat fistula [16].

In Ethiopia, there are limited numbers of hospitals that give treatment, rehabilitation, and reintegration services for obstetric fistula. Jimma University Medical Center is one of the hospitals that provide surgical repair for patients with obstetric fistula. The center supports women after surgically treated, for at least three months. The program assumes women who underwent operational repair would become comfortable with their life after treatment and would be reintegrated back to society with better confidence and self-esteem. So that the medical center provided surgical repair for several women since the service started. But, there is a paucity of information regarding the quality of life of women before and after the surgical repair. Hence, this study aimed to compare the pre-operative quality of life with that of the postoperative quality of life following successful repair of genitourinary fistula among fistula patients in the Jimma University Medical Center. The finding of this study helps the hospital managers and policymakers to consider during their planning and execution to improve the service.

## Methods

### Study area and design

An institutional-based prospective, before and after study design was employed in Jimma University Medical Centre; Jimma zone, from November 1, 2019, to October 30, 2020. The Medical Center is found in Jimma town. Jimma town is located 352 km in the Southwest of Addis Ababa, the capital. The Medical Center has a catchment population of 15 million people. It has an annual out-patient caseload of 160,000 and 106 patients had undergone fistula surgery in the last year. The Jimma University Medical Center provides services to diverse populations of three regional states; namely, Oromia, Southern Nations, Nationalities and Peoples, and Gambella regional states as well as for those belonging to a neighboring country like South Sudan.

### Study participants

All women who visited Jimma University Medical Center and presented with complaints of continuous leakage of urine per vagina following obstetric or gynecological procedures and undergone surgical repair were included in the study. Women who were seriously ill or unable to respond during the time of data collection and women with another type of fistula, such as recto-vaginal fistula, malignant fistula, radiation fistula, and/or patients with co-morbidities affecting the quality of life were excluded from the study.

### Sample size and sampling techniques

A convenient sampling technique was employed. All fistula patients, who underwent genitourinary surgical fistula repair, were included in the study. A total of 89 women with obstetric fistula were registered for surgery and provided informed consent to participate in the study. Of them,78women were completed the interview at three months. The rest of the registered were unable to complete the interview due to certain reasons. Some of them were referred to other hospitals while others left the center before three months. Women who presented with complaints of continuous leakage of urine following obstetric or gynecological procedures who were admitted to Jimma University Medical Center and eligible for genitourinary surgery were identified. Women were interviewed before the surgical intervention and three months later following the successful fistula repair.

### Data collection methods and tools

Data were collected through face-to-face interviews using a structured questionnaire. The pretested data collection instrument was adopted from the World Health Organization WHOQOL-BREF questionnaire and related relevant literature [5, 7, 16–18]. The questionnaire prepared in English was translated into the regional language Afan Oromo and retranslated back into English to ensure its consistency by the language experts. The questionnaire consists of three parts. The first part evaluates the socio-demographic characteristics; the second part evaluates the patient's subjective assessment of her quality of life and satisfaction with their state of health, while the third part assesses the four domains: physical health, psychological, social health, and environmental health. Except for the socio-demographic characteristics, all parts of the questionnaire present several questions with a five-point Likert scale for the respondent to score. All women were interviewed two times before and following the successful surgical fistula repair. Two trained diploma graduated health professionals who speak the local language were collected the data. Data collectors were trained for one day to be familiar with the data collection tool. Editing and sorting of the interview questionnaire were done to determine the completeness and consistency of data every day during the data collection.

### Data analysis

After data collection, each questionnaire was checked for completeness. Data were coded and entered into EpiData software version 3.1 and then exported to IBM SPSS version 21 for analysis. The results were presented in mean ± standard deviation (SD) and percentages. The unpaired t-test was used to compare two independent means to compare more than two independent means. The one-sample t-test was used to compare the variables in the preoperative and postoperative periods. Linear regression analysis was done for identifying determinants of the quality of life of obstetric fistula. The *P* value < 0.05 was considered statistically significant.

### Brief description of surgical method

Once the results of these preliminary investigations are available the treatment options, detail of the operation and post operative period and the possible long term squeal were explained to the woman and her husband or her families where possible. Woman and her husband/family were counseled to enable consideration of the various options before a decision can made. Informed consent for the procedure obtained and formally recorded if woman agrees to operation. Woman decides and gives her consent freely by herself. Workers in fistula clinic facilitate for admission process to hospital and the supportive care required for women awaiting surgery. Surgical techniques are employed. After the operative procedures, care is given in the fistula clinic that is mostly responsible for providing such care. Those who undergone fistula surgery need bed rest for up to 90 days after surgery depending on the type of operation and the surgeons own preference.

### Measurement

We used the WHOQOL-BREF questionnaire, a 25 items abbreviated version derived from WHOQOL Bref-100 to measure fistula patient-participants quality of life [20]. This tool generates four domains such as physical health, psychological, social relationships, and environmental health. The item scores range from 1 to 5, with a higher score indicating high quality. The domain scores were transformed into a linear scale ranging between 0 and 100 following the scoring guidelines. A mean score of less than 50 in each domain denotes poor, 50–75 indicates moderate, and greater than 75 indicates good quality of life [6, 8]. Physical health was assessed by seven questions with five-point Likert scales ranging from not at all (5) to an extreme amount (1). This scale was found to have high internal consistency (Cronbach's alpha = 0.832). These items were physical pain, the need for medical treatment to function daily work, having enough energy to work, able to get around, satisfaction with sleep, ability to perform the living activity, and satisfaction with the capacity to work. The psychological domain was measured by six items with five points Likert scale. This scale was found to have high internal consistency (Cronbach's alpha = 0.791). These items were how much they enjoy life, having a meaningful life, their ability to concentrate, satisfaction with themself, and having negative feelings. Social domain was measured by three items with five-point Likert scales. The scale had high internal consistency (Cronbach's alpha = 0.765). These questions were the support of friends, personal relation, and satisfaction with the living place. Similarly, the environmental domain was measure by seven questions with five-point Likert scales ranging from very satisfied (5) to very dissatisfied (1). These questions were the safety of life, health of the physical environment, having enough money, having the information they need, opportunity for leisure activity, satisfaction with access to health and transport. The prevalence of overall quality of life was calculated by the percent-mean formula: actual value minus potential minimum divided by potential maximum minus potential minimum [19, 21].

## Result

During the last year (12 months) of the study, 89 women had their obstetric fistula surgery repair at Jimma University Medical Center. Of them, 78 women had given informed consent and were completed the study.

## Socio-demographic characteristics

A total of 78 women with obstetric fistula were interviewed. Thirty-nine (50%) of respondents belonged in the age of 20–24 years while 18 (23.1%) were in the age group of greater than 35 years. The mean (± SD) age of the respondents was 27.23 ± (7.48) years. Thirty-five (44.9%) of respondents were divorced while 19 (24.4%) of the study participants were living in separation from their husband as a result of fistula. Regarding religion, three fourth (66.7%) of study participants were Muslim while 21 (26.9%) of respondents were orthodox in religion. More than half (56.4%) of respondents were Oromo in ethnicity and followed by Kafficho (28.2%). A majority (78.2%) of study participants were housewives in occupations. Of the study participants, 53(59.4%) were unable to read and write. The age at first marriage for a majority (70.5%) of respondents was in the age group of 15–19 years while also the age at first pregnancy for the majority (69.2%) was in the age group of 15–19 years. Fifty-four (69.2%) of respondents had given birth in the health facilities. Of the study participants, 47 (60.3%) of respondents had a history of the previous stillbirth at least once. The majority (87.2%) of study participants have no own income. Three fourth (74.4%) of the study participants had no history of antenatal care follow-up. More than half (53.8%) of respondents had given birth by SVD. The median duration of current labor was 39 h (Table 1).

**Table 1:** Sociodemographic characteristics of respondents before and after surgical repair in Jimma medical center, 2020

Characteristics	Frequency	Percentage
*Age in years*		
15–19	4	5.1
20–24	39	50.0
25–29	15	19.2
30–34	2	2.6
Greater than 35	18	23.1
*Marital status*		
Married	13	16.7
Divorced	35	44.9
Widowed	11	14.1
Separated	19	24.4
*Religion*		
Orthodox	21	26.9
Muslim	52	66.7
Protestant	5	6.4
*Ethnicity*		
Oromo	44	56.4
Amhara	3	3.8
Kafficho	22	28.2
Benchimaji	9	11.5
*Occupation*		
Housewife	61	78.2
Farmer	5	6.4
Day laborer	2	2.6
other	10	12.8
*Educational status*		
Unable to read and write	53	67.9
Read and write	11	14.1
Primary (1–8)	12	15.4
secondary and above	2	2.6
*Age at first marriage*		
15–19	55	70.5
20–24	16	20.5
25–29	7	9.0
*Age at first pregnancy*		
15–19	54	69.2
20–24	18	23.1
25–29	6	6.6
*Place of current delivery*		
Health facility	54	69.2
Home	24	30.8
*Previous stillbirth*		
No	15	19.2
Once	47	60.3
> 2 Times	16	20.5
*Having their income*		
Yes	10	12.8
No	68	87.2
*History of PNC follow-up*		
Yes	20	25.6
No	58	74.4
*Mode of delivery*		
SVD	42	53.8
C/S	11	14.1
Instrumental delivery	25	32.1

## Quality of life

The mean and standard deviation of the overall quality of life of women before the surgical repair was 58.175 ± 7.2 before the surgical repair while it has increased to71.20 ± 10.79 after the surgical repair. The difference was highly statistically significant (*P* < 0.001). Similarly, the overall satisfaction with the health status of women before the surgical repair was 22.5 ± 1.30and it has increased to 53.0 ± 0.90 after surgical repair of women with obstetric fistula. The difference was also statistically significant (*P* < 0.001).

Regarding the physical domains of quality of life before and after the surgical repair, the physical health score was 16.51 ± 5.27 before the surgical repair and it has increased to 21.77 ± 5.38 after the surgical repair. The result showed, statistically there is a significant difference in the mean value of pre and post-operative (*P* = 0.000). The psychological health score was 19.06 ± 1.46 before the surgical repair while it was increased to 19.84 ± 3.21 after the operation done for the women. The result also showed, statistically there is a significant difference in the mean value of pre and post-operative surgery (*P* = 0.048). The domain of social and environmental health scores has shown a significant change after the surgical procedure. The mean score of the social domain was 5.19 ± 1.34 before the surgical repair while it was increased to 7.13 ± 3.67 after the surgical repair. The result showed there is a significant statistical difference in the mean value of preoperative and postoperative. The mean score of the environmental domain was 17.41 ± 2.89 before the surgical repair while it was increased to 21.65 ± 4.04 after the surgical repair. The result showed there is a significant difference in the mean value of pre-operative and post-operative (*P* = 0.000) (Table 2).

**Table 2:** Quality of life of respondents before and after surgical repair in Jimma medical center, 2020

	Before	After	t	df	Sig. (2-tailed)	Mean difference	95% confidence interval of the difference	
							Lower	Upper
*One-sample test*								
Physical health	16.51 ± 5.27	21.77 ± 5.38	8.529	75	0.000	5.26632	4.0363	6.4964
Psychological	19.06 ± 1.46	19.84 ± 3.21	2.016	68	0.048	.78058	0.0080	1.5531
Social	5.19 ± 1.34	7.13 ± 3.67	4.611	75	0.000	1.94158	1.1027	2.7804
Environmental	17.41 ± 2.34	21.65 ± 4.04	9.155	75	0.000	4.24789	3.3236	5.1722
Overall satisfaction	22.5 ± 1.30	53.0 ± .90	7.458	66	0.000	1.250	2.896	2.166
The overall quality of life	58.17 ± 7.2	71.20 ± 10.79	10.033	68	0.000	13.03290	10.4408	15.6250

### Predictors of quality of life score

All variables of quality of life were entered into a linear regression model and identified factors associated with quality of life. The model explains 38.2% of the variance (R square = 0.382, *P* < 001). From all predictors: age at first marriage, satisfaction with support from friends, satisfaction with themselves, satisfaction with a capacity to work, able to concentrate, and satisfaction with the personal relationship were statistically significant factors associated with the quality of life.

Holding other variables constant, the age at first marriage was directly related to the quality of life. As the age at first marriage increased one unit, the quality of life increased by 1.522 [β = 1.522, (95% CI: 0.471, 2.573)]. Women who underwent surgical repair had 4.552 units more satisfaction with the support from their friends than before undergoing surgical repair [β = 4.552, (95% CI: 3.338, 5.766)]. Women who underwent surgical repair had 3.57 units more satisfaction with themselves than before undergoing surgical repair [β = 3.57, (95% CI: 2.413, 4.727)]. As women undergo a surgical repair, the satisfaction of women in their capacity to work certain activities had increased by 3.868 units [β = 3.868, (95% CI: 2.668, 5.067)]. As a woman with obstetric fistula gets surgical repair, the ability of women to concentrate on certain things had increased by 3.187 units (β = 3.187, (95%CI: 1.560, 4.814)). Surgical repair of a fistula increases the satisfaction of the personal relationship of women. As a woman undergo surgical repair, the satisfaction with her relationship had increased by 4.137 units [β = 3.187, (95% CI: 2.986, 5.288)]. The social score was directly related to the quality of life (β = 1.908), that is as a woman underwent a surgical repair, the social aspect of a woman had increased by 1.908 (95% CI: 1.400, 2.417)). Similarly, the psychological and physical health dimension score was directly related to the quality of life, that is as the psychological score increased a unit quality of life score increased by 2.584 [β = 2.584, (95% CI: 2.062, 3.106)]. As physical health increased a unit quality of life increased by 1.824 [β = 1.824, (95% CI: 1.570, 2.079)] (Table 3).

**Table 3:** Logistic regression analysis about factors associated with quality of life among fistula patients undergone surgical repair, South West Ethiopia, 2020

Model	Unstandardized coefficients		Standardized coefficients	t	Sig	95.0% confidence interval for B	
	B	SE	Beta			Lower Bound	Upper Bound
(Constant)	48.175	7.546		6.384	0.000	33.112	63.237
Age at first marriage	1.522	0.527	0.333	2.891	0.005	0.471	2.573
Unable to read and write	1						
Read and write	0.160	1.415	0.014	0.113	0.910	− 2.664	2.985
Formal education	2.014	1.349	0.179	1.493	0.140	− 0.678	4.706
Need medical treatment to function daily life	1.119	0.990	0.137	1.130	0.263	− 0.858	3.096
Enjoying life	− 0.349	1.061	− 0.040	− 0.329	0.743	− 2.466	1.769
Satisfaction with support from a friend	4.552	0.608	0.675	7.484	0.000	3.338	5.766
Satisfaction with your self	3.570	0.580	0.6011	6.159	0.000	2.413	4.727
Satisfaction with your capacity to work	3.868	0.601	0.618	6.436	0.000	2.668	5.067
Able to concentrate	3.187	0.815	0.431	3.909	0.000	1.560	4.814
Satisfaction with the personal relationship	4.137	0.577	0.659	7.175	0.000	2.986	5.288
Social health dimension score after	1.908	0.255	0.675	7.486	0.000	1.400	2.417
Psychological dimension score after	2.584	0.261	0.770	9.883	0.000	2.062	3.106
Physical health dimension score after	1.824	0.127	0.868	14.309	0.000	1.570	2.079

^a^Dependent variable: overall quality of life

## Discussion

Following the surgical repair, there is an improvement in the overall quality of life by 13% and the overall satisfaction to their health was improved by 50%. This result was in line with a study done in India in which the overall quality improvement by 85% following the surgical repair [6]. In our case, the project only gives medical services to fistula patients. In another part of fistula centers, social and psychological integration have given in addition to medical services. This might be the reason why there is a slight improvement. From the domains of quality of life psychological domain was not showed large improvement after surgical repair. Before the surgery, the quality of life was 19.06 while it was 19.84 after surgical repair. A substantial portion of women was not satisfied with their relations, the support they get from their friends, and the condition of their living places which confirms the occurrence of psychological trauma on women. From the environmental health domains, the majority of women have no money to meet their needs, able to get enough information in their daily life, and have no opportunity for leisure activities. The possible explanation for this might be women faced poor support. In this regard, Jimma University medical center provides some financial support, but this support is not enough for them. Primarily, women with fistula need assistance from husband, family, and community members. On contrary, they divorced because of the fistula and become psychologically ill, and lack the necessary support. In our study, about 45% of women had divorced and about 24% were living separated from their husbands as a result of fistula. The majority of women who participated in this study were illiterate. Education has a direct relationship with awareness. It has been known that lack of awareness affects the health-seeking behavior of women. In this study, the majority of women married and got pregnant in the age group of 15–19, which may indicate the prevalence of early marriage in the country which contributes a lot to the incidence of fistula. Early pregnancy and home delivery expose women to the risk of fistula [10]. Obstructed labor contributes high to the case of fistula. In this study, the majority of women who were with obstetric fistula and participated in this study gave birth at their home. Place of delivery was a significant factor associated with the problem encounter during the delivery [22].

In our study, there is a significant change in the physical health domain among women who underwent surgical repair at Jimma University Medical Center (*P* < 001). This result is explained by the fact that relief from physical pain, physical improvement, satisfaction with their sleep, and their day-to-day work. This result is in line with a study done in India, Nigeria, and another part of the country [6, 15, 19]. Similarly, the social health domain showed significant improvement after a woman gets a surgical repair. Rejection from their husband, friends, families and feeling dissatisfied in their condition of the living place was the manifestation of social impacts. Statistically, there is a significant improvement in the social domain after the surgical procedure (*P* < 0.001). The result of the present study is in line with the study done in other parts of the world in which surgical repair improves the social aspect of women [6, 8, 15, 19].

## Conclusion

In conclusion, the overall quality of life was improved after successful surgical repair. All domains of quality of life have shown significant improvement after the surgical repairs. The psychological related factors showed little improvement compared to other domains. Physical health and social health dimensions showed more change than other dimensions. Quality of life was influenced by satisfaction with support from friends, the capacity to work, and the ability to concentrate to do certain activities. It is recommended that the medical center and other concerned parties should implement psychological and social rehabilitation and reintegration mechanisms in addition to medical interventions to improve the quality of life of women with fistula.

## Data Availability

The data collected for this study can be obtained from the first author based on a reasonable request at the email: tilahunfufa@gmail.com.
